# Trans-Atlantic analysis of gender representation in general thoracic surgery: Challenges permeate the academic community

**DOI:** 10.1016/j.xjon.2025.06.006

**Published:** 2025-06-12

**Authors:** Rajika Jindani, Debora Brascia, Albert Dweck, Javeria Tariq, Justin Olivera, Amanda Ghanie, Jorge Humberto Rodriguez-Quintero, Mara B. Antonoff, Brendon M. Stiles, Cecilia Pompili

**Affiliations:** a Department of Cardiothoracic Surgery, Montefiore Medical Center/Albert Einstein College of Medicine, Bronx, NY; b Department of Biomedical Sciences, Humanitas University, Milan, Italy; c Department of Thoracic Surgery, IRCCS Humanitas Research Hospital, Rozzano, Milan, Italy; d Department of Thoracic Surgery, St James's University Hospital, Leeds, United Kingdom; e Department of Thoracic Surgery, University of Texas MD Anderson Cancer Center, Houston, Tex; f Department of Thoracic Surgery, Hull University Teaching Hospitals, Hull, United Kingdom; g Department of Thoracic Surgery, York and Hull Medical School, University of Hull, Hull, United Kingdom

**Keywords:** gender disparities, thoracic surgery, diversity, equity, and inclusion, workforce analysis

## Abstract

**Objective:**

The underrepresentation of women in thoracic surgery has been well described worldwide. Women can serve as role models for trainees and advance their careers through academic appointments, leadership positions, and involvement in thoracic societies. We aimed to characterize differences between representation of women in thoracic surgery in the United States and Europe.

**Methods:**

A cross-sectional study was conducted using publicly available data for hospitals with 30 general thoracic-track training programs in the United States and Europe from December 2023 to May 2024. Membership data for national/international societies were obtained directly from respective organizations.

**Results:**

Among 30 US institutions with dedicated general thoracic surgery training tracks, women comprised 17.7% (102 out of 475) of faculty, compared with those of 30 general thoracic surgery centers in 8 European countries, where women comprised 29.5% (79 out of 268) of faculty. Of programs with available data, 26.7% (8 out of 30) had women as thoracic surgery program directors in the United States and 13% (4 out of 30) in Europe. Regarding societal membership in the General Thoracic Surgical Club (United States) and European Society of Thoracic Surgeons (Europe), women were well represented as trainee members (United States, 39.2% [20 out of 51] vs Europe, 46.1% [113 out of 245]; *P* = .367), but comprised a lower proportion of active/senior members (United States, 12.9% [45 out of 349] vs Europe, 19.2% [283 out of 1474]; *P* = .006).

**Conclusions:**

We identified universal disparities in the representation of women in faculty appointments, leadership positions, and membership in professional societies. Efforts to address imbalances may benefit from shared experiences and initiatives, aiding resident recruitment and career advancement for women thoracic surgeons while fostering diversity, equity, and inclusion on a global scale.

**Figure:**
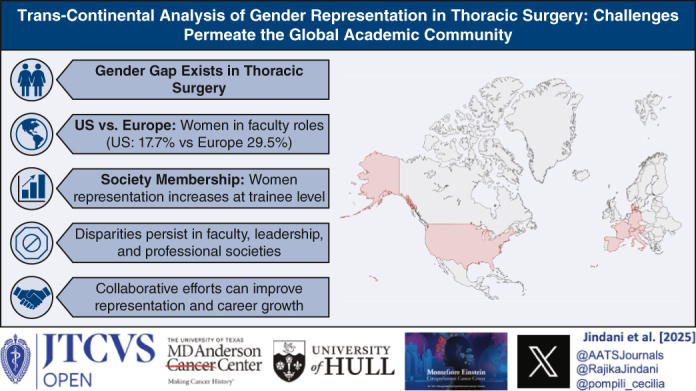
Significant disparities in representation of women in thoracic surgery exist globally.

**Central Message**

Global disparities in the representation of women in thoracic surgery persist, highlighting barriers to leadership and equity. Collaborative efforts are essential to foster diversity and inclusion.

**Perspective**

The underrepresentation of women in thoracic surgery reflects an issue transcending borders, with stark disparities in leadership and senior roles. Although regions show progress at trainee levels, barriers, such as mentorship gaps, hinder advancement. Addressing these challenges requires tailored strategies, global collaboration, and systemic changes to build an inclusive, diverse workforce.

The underrepresentation of women in thoracic surgery is a well-documented issue with significant global implications.^1^, ^2^, ^3^, ^4^, ^5^, ^6^, ^7^, ^8^ In the United States, women have composed <10% of practicing cardiothoracic surgeons, underscoring a persistent gender imbalance within the field.^9^ This disparity is not limited to any single country; rather, it is a widespread challenge that affects regions worldwide.^10^^,^^11^ Understanding these regional variations and how they influence training environments is essential because they may highlight specific barriers or successful strategies that could inform recruitment and retention efforts in thoracic surgery.

The advancement of thoracic surgery is closely tied to the inclusion of women in leadership roles, academic appointments, and professional societies.^12^, ^13^, ^14^ Women who occupy these positions not only serve as vital role models for the next generation of surgeons but also contribute diverse perspectives that foster innovation and progress in both clinical practice and academic research.^15^^,^^16^ Their presence is essential for driving progress and ensuring that the field reflects the diversity of the communities it serves.

Women in academic departments play a pivotal role in inspiring and mentoring aspiring thoracic surgeons.^17^ Their leadership challenges entrenched stereotypes, demonstrating that success in this demanding specialty is achievable for women. Women faculty members bring invaluable insights and experiences that can significantly influence the development of trainees.^18^ Additionally, women in academia can offer mentorship tailored to the specific challenges faced by women trainees, such as balancing career demands with personal responsibilities and overcoming implicit biases.^19^ This supportive guidance not only helps foster professional growth but also contributes to a more inclusive and dynamic field, encouraging more women to pursue and thrive in thoracic surgery.

This study aims to conduct an analysis of the representation of women in thoracic surgery in academic departments associated with training programs across the United States and Europe. This global assessment allows for the identification of best practices from regions where women are more equitably represented and to inform the development of region-specific interventions. By focusing exclusively on general thoracic surgery training programs, we ensure a fair comparison between the United States and Europe, given that European training paradigms typically separate thoracic and cardiac surgery departments, whereas the United States training is combined. Through an analysis of the proportions of women in academic faculties, leadership positions, and professional societies, this research seeks to identify patterns, disparities, and areas that demand universal targeted interventions, particularly in relation to fostering training environments welcoming to persons from all backgrounds. Ultimately, the findings will contribute to the ongoing global dialogue on diversity, equity, and inclusion within general thoracic surgery, providing actionable insights that can help promote the growth and advancement of women surgeons worldwide.

## Materials and Methods

### Study Design and Data Sources

This study employed a cross-sectional design to analyze the representation of women in general thoracic surgery departments associated with training programs within the United States and Europe. To capture all faculty involved in the education and mentorship of general thoracic track trainees, US programs included both cardiac and thoracic faculty, whereas European programs focused exclusively on thoracic faculty due to the differences in departmental structure. In the United States, general thoracic trainees are often mentored by a mix of cardiac and thoracic attendings, whereas in Europe, the training paradigm and available mentors differ. This study compares general thoracic surgery faculty and leadership in the United States and Europe, contextualizing findings within broader international disparities. In the United States, both cardiac and thoracic faculty were included, reflecting the integrated structure of general thoracic training programs, where mentorship spans both subspecialties. Data were collected from publicly available sources and cross-referenced by contacting institutions directly, as well as supplemented with information directly obtained from available national and international thoracic surgery societies. The study period was December 2023 to May 2024.

For the United States, we identified institutions with general thoracic surgery training tracks, including both traditional and integrated programs, through the Accreditation Council for Graduate Medical Education database and individual institutional websites. A total of 30 institutions were included in the analysis. Data on academic faculty, including the number and gender of faculty members, were extracted from departmental and institutional websites. Information on leadership positions, particularly those that directly influence trainee experience, such as thoracic surgery program directors, thoracic surgery chiefs, and surgery chairs, was also collected. For Europe, thoracic surgery centers were identified from the European Society of Thoracic Surgeons and other relevant national societies. A total of 30 thoracic surgery centers across 8 European countries were included. Similar to the US data collection, information on academic faculty and leadership positions was obtained from publicly accessible sources, including institutional websites, national databases, and by directly contacting institutions.

Membership data were obtained directly from national and international general thoracic surgery societies, with active and senior members included, as well as trainee members to evaluate differences in gender representation by level. This included the General Thoracic Surgical Club (GTSC), the European Society of Thoracic Surgeons (ESTS), and the Society of Thoracic Surgeons (STS). These organizations were chosen because both GTSC and ESTS are general thoracic societies for their respective regions and STS is a large international professional society. Membership categories were analyzed to compare the proportions of women among the society membership classifications: trainee members (students, residents, and fellows) and active surgeons/senior members. Due to limitations in publicly available data, STS membership figures were included for descriptive purposes only and were not incorporated into statistical analyses or comparative tables, as granular data comparable to ESTS and GTSC were unavailable. Limited granularity precluded formal inclusion in Table 5 or comparative statistical analyses.

**Table 5:** Breakdown of membership in General Thoracic Surgical Club and European Society of Thoracic Surgeons in 2023

Organization	Women	Men	*P* value
Trainee/candidate members			.367
General Thoracic Surgical Club, n = 51	20 (39.2)	31 (60.8)	
European Society of Thoracic Surgeons, n = 254	113 (46.1)	141 (55.5)	
Active/senior members			.006∗
General Thoracic Surgical Club, n = 349	45 (12.9)	304 (87.1)	
European Society of Thoracic Surgeons, n = 1474	283 (19.2)	1191 (80.8)	

Values are presented as n (%). ∗Indicates statistically significant result.

Comparative analyses were conducted between the United States and Europe regarding the representation of women in thoracic surgery. We performed χ^2^ analyses and *z* tests for proportions to assess the statistical significance of differences in the proportions of women in various roles, including academic faculty, leadership positions, and societal memberships. All statistical tests were 2-sided Statistical analysis was performed using SPSS version 28 (IBM Corporation). All data used in this study were obtained from publicly available sources or were provided by professional societies upon request. Because this study did not involve any human subjects or patient data, formal ethical approval was not required.

## Results

### Women Representation

**Figure 1:**
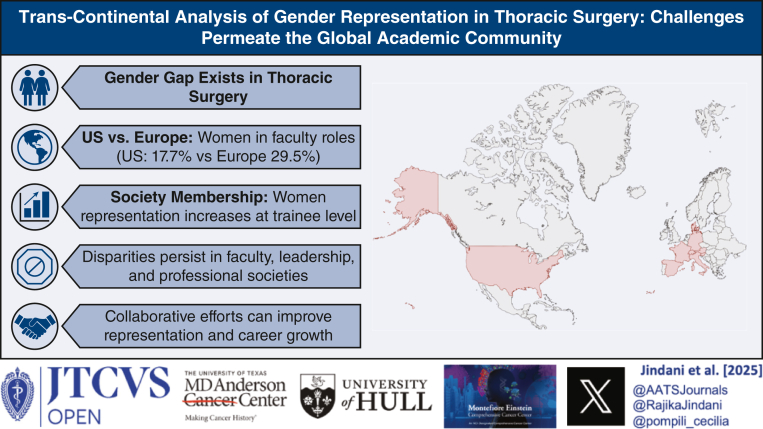
Significant disparities in representation of women in thoracic surgery exist globally.

Table 1 compares the overall gender representation of surgeons identified as faculty in departments between the United States and Europe. To accommodate differences in training paradigms, US data included cardiac and thoracic faculty, whereas European data were specific to thoracic surgery for valid regional comparisons while acknowledging integration in US practice. The data shows a significant difference in the distribution of men and women staff surgeons between the 2 regions (*P* < .001). In the United States, 82.3% of the surgeons were men, and 17.7% were women. Conversely, in Europe, 70.5% of the surgeons were men, and 29.5% were women (Figure 1).

**Table 1:** Number of women surgeons represented in departments in the United States versus Europe

Category	United States (n = 577)	Europe (n = 268)	*P* value
Men	475 (82.3)	189 (70.5)	<.001∗
Women	102 (17.7)	79 (29.5)	

Values are presented as n (%). ∗Statistically significant *P* value.

Table 2 presents the breakdown of gender representation across different regions of the United States and academic ranks within the field. Among the regions, the distribution of men and women surgeons did not show a statistically significant difference (*P* = .132). Specifically, the Northeast region had 18.2%, the Midwest had 14.5%, the South had 16.1%, and the West had 28.1% women. In contrast, gender distribution across academic ranks showed a significant difference (*P* < .001). Among assistant professors, 27.8% were women, for associate professors, 17.1% were women, and for full professors, only 8.2% were women.

**Table 2:** Gender representation breakdown in the United States by region and ranking

Variable	Men (n = 475)	Women (n = 102)	*P* value
Region			.132
Northeast, 225 (39)	184 (81.8)	41 (18.2)	
Midwest, 152 (26.3)	130 (85.5)	22 (14.5)	
South, 143 (24.8)	120 (83.9)	23 (16.1)	
West, 57 (9.9)	41 (71.9)	16 (28.1)	
Academic rank			<.001∗
Assistant professor, 176 (30.9)	127 (72.2)	49 (27.8)	
Associate professor, 111 (19.5)	92 (82.9)	19 (17.1)	
Full professor, 171 (30.1)	157 (91.8)	14 (8.2)	
Unknown/no ranking	91 (82)	20 (18)	

Values are presented as n (%). ∗Statistically significant *P* value.

A subgroup analysis of the breakdown of all US faculty by cardiac and general thoracic surgery is shown in Table 3, which highlights gender distribution and academic rank across general thoracic versus cardiac surgery faculty in US programs. Differences in overall faculty gender distribution (29.5% in Europe vs 17.7% in the United States) appear largely driven by cardiac surgery representation, as gender distribution among general thoracic surgeons in the United States is more comparable to Europe, yet still significantly lower (21.5%).

**Table 3:** Subgroup analysis of United States faculty by cardiac and general thoracic surgery

Category	Men (n = 475)	Women (n = 102)	*P* value
Gender			.08
Cardiac surgery, n = 377	318 (66.9)	59 (57.8)	
Thoracic surgery, n = 200	157 (33.1)	43 (42.2)	
General thoracic surgery, academic rank			.133
Assistant professor, 59 (29.6)	41 (26.3)	18 (41.9)	
Associate professor, 36 (18.1)	30 (19.2)	6 (14.0)	
Full professor, 53 (26.6)	46 (29.5)	7 (16.3)	
Unknown/no ranking, 51 (25.6)	39 (25.0)	12 (27.9)	
Cardiac surgery, academic rank			<.001
Assistant professor, 117 (31.6)	86 (27.7)	31 (52.5)	
Associate professor, 75 (20.3)	62 (19.9)	13 (22.0)	
Full professor, 118 (31.9)	111 (35.7)	7 (11.9)	
Unknown/no ranking, 60 (16.2)	52 (16.7)	8 (13.6)	

Table 4 presents the breakdown of gender representation across different regions of Europe and academic ranks within the field. In the analyzed areas, there were no statistically significant differences by region in men and women faculty (*P* = .905). However, the number of academic members across all European nations revealed a marked increase in the number of men (76.5%) compared with women (23.5%) faculty.

**Table 4:** Gender representation breakdown in Europe by region and ranking

Variable	Men (n = 189)	Women (n = 79)	*P* value
Region			.905
Eastern Europe, 12 (4.5)	9 (75)	3 (25)	
Northern Europe, 10 (3.7)	7 (70)	3 (30)	
Southern Europe, 110 (41.0)	75 (68.2)	35 (31.8)	
Western Europe, 136 (50.7)	98 (72.1)	38 (27.9)	
Academic position			
All ranks, 102 (38)	78 (76.5)	24 (23.5)	

### Academic Faculty Representation

**Figure 2:**
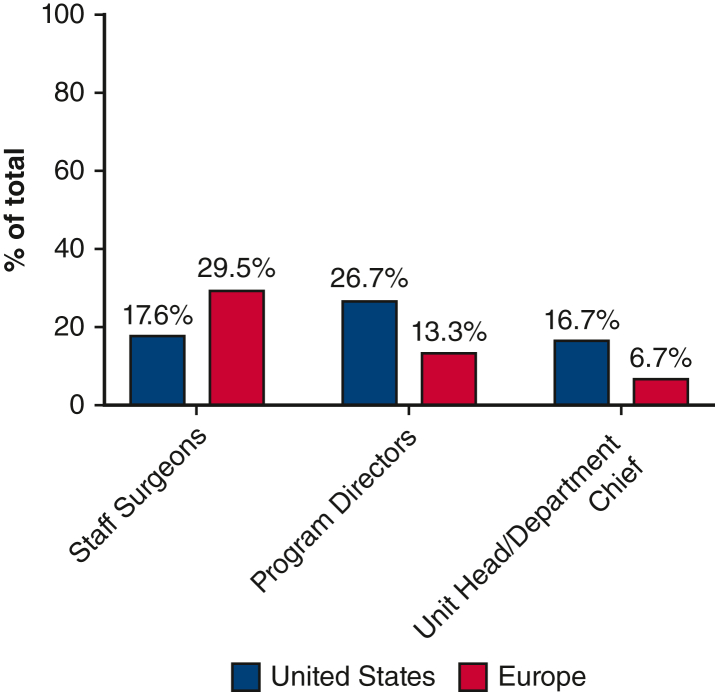
The representation of women as staff surgeons in departments affiliated with thoracic training programs, program directors of general thoracic track training programs, and unit head or departmental chiefs.

In the United States, among the total academic faculty positions in these programs, women comprised 17.6% (82 out of 466). When examining leadership roles within these US institutions, women held 26.7% (8 out of 30) of thoracic surgery program director positions, 16.7% (5 out of 30) of thoracic surgery chief positions, and 10% (3 out of 30) of surgery chair positions. In Europe, data from 30 thoracic surgery centers across 8 European countries showed women comprised 29.5% (79 out of 268) of staff positions with 13.3% (4 out of 30) of these centers having women serving as thoracic surgery program directors, and 6.7% (2 out of 30) had women as thoracic surgery unit heads of departments (Figure 2).

### Societal Membership Representation

**Figure 3:**
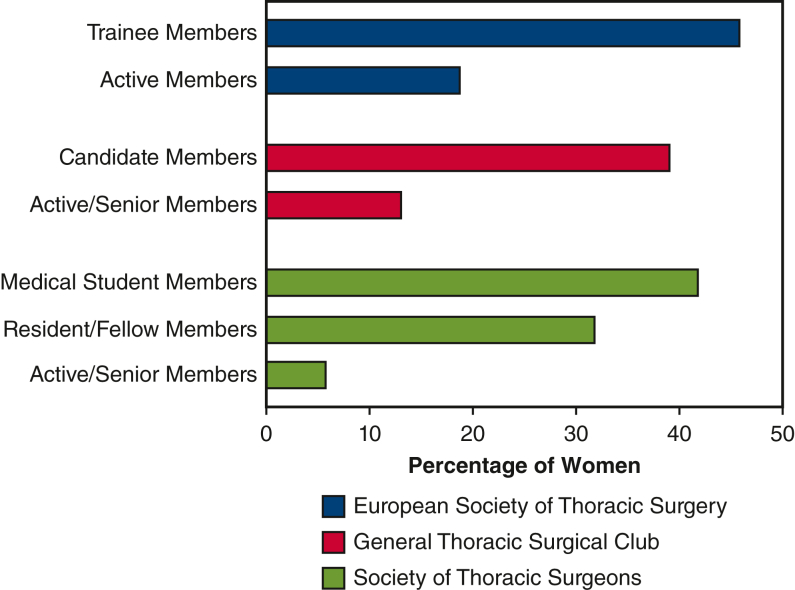
Representation of women in national/international thoracic societies based on membership level in 2023.

The study also examined the representation of women within national and international thoracic surgery societies, comparing the United States and Europe. Membership data revealed a more nuanced picture of gender representation within these professional organizations (Figure 3 and Table 5). In the GTSC, women comprised 39.2% (20 out of 51) of trainee members within thoracic surgery societies. However, inactive or senior members, women accounted for only 12.9% (45 out of 349) of the membership. Within the ESTS, women comprised 46.1% (113 out of 245) of trainee members (ESTS vs GTSC trainees; *P* = .367). The representation of women among active or senior members was lower, although still higher than in the United States, with women making up 19.2% (283 out of 1474) of this group (ESTS vs GTSC active/senior members; *P* = .006). Finally, within STS, which is the largest cardiothoracic society globally, resident and fellow members were 32%, whereas surgeons and senior members were 6% women.

## Discussion

This study underscores the persistent underrepresentation of women in thoracic surgery, reflecting deep-seated challenges that extend beyond individual institutions or countries. Despite some progress in increasing the number of women trainees entering the field, the transition from trainee to senior roles, particularly in academia and leadership, remains a significant hurdle.^9^ The significant gaps in the representation of women in academic faculty, leadership roles, and professional societies speak to the broader culture of surgery as a traditionally male-dominated field.

Our study, which compares the representation of women in general thoracic surgery across the United States and Europe, sheds light on these disparities and prompts critical reflection on why these gaps persist and how they might be effectively addressed. Despite some advances, women remain underrepresented in roles tied to academic influence such as guideline panels, conference involvement, editorial boards, and leadership.^1^^,^^2^^,^^7^^,^^8^ These deficiencies, combined with limited mentorship and systemic bias, may hinder advancement even when representation at entry levels improves.

### American Perspective

This study highlights the persistent gender disparities in thoracic surgery, particularly in the United States. The leaky pipeline phenomenon is well documented in medicine, describing the declining representation of women at each career stage.^20^ Multiple factors contribute to this attrition, including structural barriers and work-life balance challenges. Surgical trainees are guaranteed 12 weeks of unpaid leave for medical and family reasons, with recent changes by the Accreditation Council for Graduate Medical Education mandating 6 weeks of paid parental leave for all trainees.^21^ This aligns with efforts to create a more inclusive training environment, although efforts by the Association of Women Surgeons push for further reform to support work-life balance, reduce burnout, and encourage more women to pursue surgical careers.^21^ Barriers may include implicit biases, lack of mentorship, and the challenges of balancing career advancement with personal responsibilities, particularly in a specialty as demanding as thoracic surgery.^22^

Our findings also underscore the importance of professional societies in shaping the trajectory of women's careers in thoracic surgery. These societies are not just networks for sharing research and clinical practices; they are gatekeepers for leadership opportunities, visibility, and professional development. The lower representation of women among active and senior members, particularly in the United States, suggests that more needs to be done to ensure that these societies are inclusive and supportive environments where women can thrive.^23^^,^^24^ This could include targeted mentorship programs, leadership training specifically for women, and active efforts to dismantle the subtle and overt biases that may pervade these organizations. Organizations, such as the Women in Thoracic Surgery, have been instrumental in mentoring and fostering an environment for women cardiothoracic surgeons in the United States.^25^

### European Perspective

The European perspective on gender diversity within thoracic surgery bears a resemblance to the American perspective, underscoring the existence of a notable gender gap. Although Europe exhibits a higher proportion of women thoracic surgeons than the United States, fewer women occupy senior and leadership positions. This phenomenon may be attributed to several factors, including implementing some working-life balance initiatives as shorter work hours in Europe (60 h/wk vs 80 h/wk in the United States) and longer maternity leave (16 weeks compared with 6 weeks in the United States).^26^ The extended leave period for both men and women allows for better recovery and bonding time, easing the pressure on trainees to return to work quickly.^27^^,^^28^

Prior studies have elucidated the challenges encountered by women in their surgical careers, encompassing lower rates of completing surgical residencies, obtaining board certification, and advancing professionally.^29^ Despite recruitment and mentorship efforts, challenges in residency support, leadership roles, work-life balance, and pay equity persist in Europe, particularly regarding mentorship and support networks to combat gender biases in surgery. Initiatives addressing gender bias include Women in Surgery in Italy, Cirujanas y Líderes in Spain, the German Association of Women Surgeons, and Women in Thoracic Surgery in France.

### Call to Action

Looking ahead, the thoracic surgery community must take a proactive stance in addressing these disparities.^30^^,^^31^ This will require a multifaceted approach that includes changes at the institutional level, such as more transparent and equitable hiring and promotion practices, as well as broader cultural shifts within the field. Institutions must prioritize creating an environment where diverse talents are recognized and nurtured, and where the unique challenges faced by women are acknowledged and addressed. The findings highlight the need for targeted strategies to support the advancement of women in thoracic surgery.^32^ This could include mentorship programs, leadership training, and policies that promote work-life balance, all of which could help to retain and advance women within the specialty.^33^^,^^34^

Several systemic factors continue to act as barriers to advancement for women in thoracic surgery. These include possible implicit bias in hiring and promotion decisions, limited access to high-quality mentorship, and a lack of sponsorship opportunities, particularly those that facilitate visibility and leadership within academic and professional organizations.^35^ Although programs such as Women in Thoracic Surgery (United States) and national-level initiatives in Europe have provided vital support, they are often underresourced or reach only a subset of those who could benefit.^24^^,^^25^ Expanding access to formal mentorship programs that also address gender bias, leadership development, and work-life integration could facilitate greater retention and progression of women in the field.^36^

Additionally, differences in access to research funding, grant support, and protected academic time may also contribute to observed disparities. Workplace culture and lack of institutional flexibility in training and career progression also represent significant challenges.^37^^,^^38^ Even in settings where parental leave is more generous, cultural expectations around working hours, availability for conferences and leadership roles, and the undervaluing of caregiving responsibilities can act as deterrents to women's advancement. Moreover, intersectional barriers, including race, ethnicity, and socioeconomic background, may further hinder advancement, though data in thoracic surgery is currently limited in this area. These compounding barriers must be examined in future work to ensure a fully inclusive and equitable approach to workforce development.

Fostering a global dialogue on best practices for promoting gender equity in thoracic surgery could help to address these disparities and support the development of a more diverse and inclusive global community in this field.^39^ Further study on how work hours, parental leave, and institutional support structures differ between the United States and European countries could inform how these potentially influence retention and leadership representation.^40^ For instance, some countries offer longer paid parental leave and formal work-hour restrictions, which may facilitate career sustainability. A transcontinental analysis could help identify high-impact strategies and inform evidence-based reforms that enhance equity and career advancement opportunities globally.

This article and its methodology have limitations. The training paradigms in the United States and Europe differ, with cardiac surgery being integrated with general thoracic in the United States, whereas in Europe, cardiac and thoracic surgery have distinct training tracks in some countries. Because the purpose of the article is to assess mentorship for trainees, the faculty considered (general thoracic vs cardiac) varies between regions and future study will look more into the differences between European countries. The data collection method included utilizing institutional websites to collect data, some of which may be inaccurate at the time of collection. Additionally, Europe has a diverse variety of training paradigms across different countries, which could not all be captured within the scope of this article.

Due to the cross-sectional nature of this study and lack of available information online, we were unable to assess trends over time and career progression. Longitudinal data would provide a more rigorous basis for evaluating differential career progression and better support conclusions related to the leaky pipeline phenomenon. Variables such as time since board certification or duration at each academic rank were not consistently available across all institutions. Some of the data we received from universities and professional societies were deidentified, which prevented analysis of individual career trajectories. Nevertheless, we agree that metrics such as time-in-rank, attrition, and the proportion of women at each level of academic and society leadership are essential for future investigation and could substantially enrich our understanding of structural barriers in thoracic surgery.

Factors such as socioeconomic disparities and differences in high versus low-income countries were also not included. Although this analysis focuses on the United States and Europe, these findings offer insights into broader patterns of underrepresentation globally. However, given the study's regional scope, we caution against overgeneralization to a global context. Because the aim of the article was to assess representation of women in academic departments, private practice surgeons were largely not included. Direct comparisons of policies related to work environment and leave practices by location were not made in this article due to the large variability in this study area; however, this is an important topic for future study.

## Conclusions

Although significant disparities in gender representation in thoracic surgery exist across the United States and Europe, there are also areas of opportunity. By learning from the experiences and strategies of different regions, and committing to sustained efforts to promote gender equity, the thoracic surgery community can move toward a future where women are not just participants in the field, but leaders and innovators who help shape its direction and improve patient care outcomes while enhancing diversity, equity, and inclusion on a global scale.

## Conflict of Interest Statement

Dr Stiles reports personal honoraria or consulting fees from AstraZeneca, Bristol Myers Squibb, Genentech/Roche, Regeneron, Pfizer, Gala Therapeutics, Medtronic, Merck, and Genentech/Roche; research funding from Bristol Myers Squibb Foundation; patents, royalties, and other intellectual property related with a therapeutic antibody targeting ART1, an extracellular mono-ADP ribosyltransferase, for the treatment of cancer patent application filed; and relationships with the Lung Cancer Research Foundation, Lungevity, and SIGA Technologies. All other authors reported no conflicts of interest.

The *Journal* policy requires editors and reviewers to disclose conflicts of interest and to decline handling or reviewing manuscripts for which they may have a conflict of interest. The editors and reviewers of this article have no conflicts of interest.
